# Use of segmented CT transmission map to avoid metal artifacts in PET images by a PET-CT device

**DOI:** 10.1186/1471-2385-5-3

**Published:** 2005-06-14

**Authors:** Siroos Mirzaei, Michel Guerchaft, Christopher Bonnier, Peter Knoll, Michel Doat, Peter Braeutigam

**Affiliations:** 1 Centre National PET, Clinique Ste. Thérèse, Luxembourg; 2 Wilhelminenspital, Institute of Nuclear Medicine, Vienna, Austria; 3 Philips Medical Systems, BeNeLux

## Abstract

**Background: ** Attenuation correction is generally used to PET images to achieve count rate values independent from tissue densities. The goal of this study was to provide a qualitative comparison of attenuation corrected PET images produced by a PET-CT device (CT, 120 kV, 40 mAs, FOV 600 mm) with and without segmentation of transmission data (ACseg^+ ^and ACseg^-^respectively). Methods: The reconstructed images were compared to attenuation corrected images obtained with a high-energy transmission source (Cs-137 – 662 keV).

Thirty oncologic patients were studied using CT and ^137^Cs for attenuation correction. All image data were acquired using the Gemini PET-CT scanner (Philips Medical Systems). It is an open PET-CT system that consists of the MX8000 multislice CT and the Allegro PET scanner arranged in a separable configuration. Images with ACseg^+ ^and ACseg^- ^were analyzed simultaneously in coronal, sagittal and transaxial planes. Two nuclear medicine physicians reviewed the image sets. Results: The image quality in the area of metal implants was better with ACseg^+ ^than ACseg^-^, without metal induced artifacts generally observed in CT corrected images. Further the images with ACseg^+ ^were qualitatively comparable to those obtained with ^137^Cs attenuation correction. Conclusions: In case of metal implants, PET studies corrected by CT should preferably use the ACseg^+ ^method to avoid the image artifacts.

## Background

Attenuation correction (AC) is generally applied to achieve count rate values independent from tissue densities. Transmission scanning was already suggested in 1952 by Myneord [1]. The transmission scanning was used to match anatomical contours to the radionuclide distribution measured by a rectilinear scanner [2]. Different isotopes have been used to acquire transmission data as earlier I-125, I-131, Tm-170 and later Tc-99 m, I-131, Ba-133, Cs-137 and Ge-68. With the development of SPECT and PET systems the transmission scanning was used for the purpose of AC and various methods for the use of transmission data were developed [3,4]. The PET transmission scan is ideally acquired before the radiopharmaceutical is administered, but would lead to long clinical protocols which are not suitable for routine use. Today, for PET-investigations post-injection transmission scans are performed using electronically windowed rotating rod sources as Ge-68, or rotating point sources as Cs-137 or CT in PET-CT systems. The combined PET-CT systems have the advantage to provide anatomical information while CT transmission data can be used for AC of the emission scan. This results in getting structural and metabolic information in the same session and a joint report of a nuclear medicine physician and a radiologist is then sent to the referring physician. However the PET examinations corrected for attenuation by CT images may be hampered by artifacts which are not usually seen in only PET images, as respiration [5], truncation [6], CT contrast agents and metal implants [7-9]. It is known that the high atomic numbers of contrast agents or metal implants result in increased fraction of photelectric interactions, yielding increased Housfield units (HU). With the current used reconstruction algorithms, contrast can be therefore missclassified as high density bone and thus associated with an incorrect scaling factor [10]. The goal of this study was to provide a qualitative comparison of attenuation corrected PET images of patients with metal implants produced by a PET-CT device with and without segmentation of CT.

## Methods

Thirty oncologic patients with metal implants (hip prosthesis, dental implants, pacemaker) were studied using CT and Cs-137 transmission scans for attenuation correction. None of the included patients had clinical signs of local inflammation of the implants. All image data were acquired using the Gemini Dual PET-CT scanner (Philips Medical systems). The GEMINI Dual is an open PET-CT system that combines a helical dual slice CT and a 3D PET scanner equipped with its own transmission source [11]. Gantries are arranged in a separable configuration that allows the use of the 2 scanners separately when desired. The MX8000 EXP CT scanner is a dual slice system whose detector consist of 1344 Cadmium Tungstate elements. Gantry allows a patient port of 70 cm. Minimum scan time per full rotation is 0.5 s. and slice thickness can range between 0.5 mm and 10 mm. The Allegro 3D PET scanner works exclusively in 3D detection mode (no septa). It is comprised of 29 arrays of 616 GSO (gadolinium oxyorthosilicate) crystals each. Crystal dimensions are 4 × 6 × 20 mm^3^. The axial field of view is 180 mm and patient port is 63 cm. The rotating point source of Cs-137 located in the gantry allows acquisition of transmission scans in 3D detection mode. Cs-137 emits single photons with energy of 662 KeV.

Patients were scanned 1 hour after injection of 4 MBq/Kg FDG. A low dose CT helical scan was performed first (scan field of 600 mm, increment of 5 mm, slice thickness 6.5 mm, pitch of 1.5, 0.75 second per rotation, matrix 512 × 512, 120 KV, 40 mAs), followed by the Cs-137 transmission scan. The high activity of the Cs-137 source (750 MBq – half-life 30 years) and the detection of single events allows fast transmissions scan. Total duration of Cs-137 transmission scan is about 4 minutes (100 cm scan length). PET emission scan is automatically started at the end point of transmission. Emission scan consists of 8 to 11 bed positions of 3 minutes each which allows to cover 77 cm to102 cm. Randoms are online subtracted during acquisition (time-delayed events). Total acquisition time per patient varied from 30 to 40 minutes.

4 sets of data were reconstructed for each patient: no AC, AC with Cs-137 point source, AC with non-segmented CT image (Acseg^-^) and AC with segmented CT (Acseg^+^). Acquisition data were processed with standard package delivered with the system (Petview software – Philips Medical Systems). Reconstruction without AC was performed with RAMLA 2D iterative algorithm [12,13]. All reconstructions with AC were performed with RAMLA 3D iterative algorithm [12,14]. Voxel size after reconstruction is 4 × 4 × 4 mm^3^. Scatter correction is performed by fitting the tails of activity outside patient with a parabolic curve. AC with Cs-137 source is performed by segmentation of original transmission data set. Segmentation is used to divide the CT image into regions representing different tissue types (lung, soft tissue, bone). All pixels in regions corresponding to different tissues are assigned the value associated with these particular tissue types. Regions with higher attenuation coefficients than bone tissues (metal implants) are assigned to soft tissue attenuation coefficient. Natural variations in densities are reflected by merging [15,16] segmented image with the measured attenuation map that is first corrected such that the 'average' values of lung tissue's and soft tissue's attenuation coefficients are equal to nominal values set in reconstruction protocol file. Merging is controlled by lung and soft tissues attenuation coefficients (user defined uniformity parameters). With values set to 1.0, only the segmented image is used and no merging is performed. With values set to 0.0, only an over-smoothed mean-adjusted image of measured transmission is used. Intermediate values result in a mix between the two. Default values were used (0.50 for lungs and 1.0 for soft tissues). Computation of AC with low dose CT data requires first the down-sampling of CT scan to PET matrix size and pixel size. Then CT truncation compensation is applied to compensate artifacts resulting in using a FOV of 600 mm [17]. Resolution matching is then performed by smoothing CT images. Gaussians filters are used with FWHMs that match PET resolution in axial and transaxial directions. CT images with pixels in Hounsfield Units (HU) are then converted to linear attenuation coefficients at 511 KeV [18,19]. Converted CT images are segmented to perform AC by dividing maps into regions representing tissue types, as described before. Images with ACseg^+ ^and ACseg^- ^were displayed simultaneously in three different planes (coronal, sagittal, transaxial). Two nuclear medicine physicians reviewed the data sets with a specific multi-modality registration software (Philips Syntegra). PET and CT images can be visualized independently as well as registered on the same screen in coronal, sagittal and transverse planes. Layout can be customized and different presets are available to scale the CT images (lungs, soft tissues, bone, abdomen ...). Segmented transmissions scans obtained with Cs-137 and CT were also compared.

## Results

**Figure 1:**
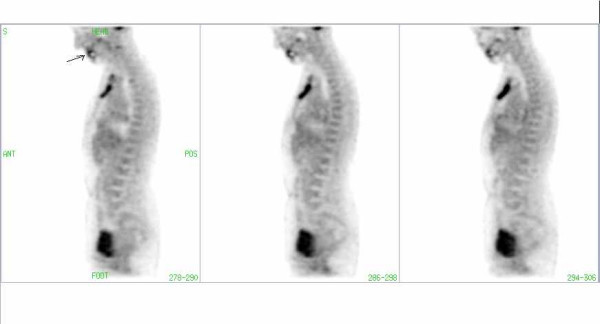
FDG-PET image of a patient with a hypermetabolic retrosternal mass. Artifact (arrow) due to a dental implant in CT corrected images without segmentation.

**Figure 2:**
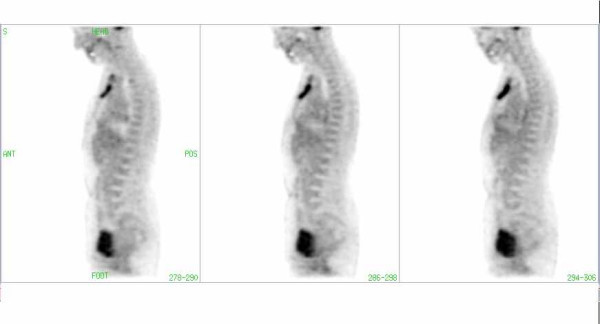
The same patient as in Fig. 1: The Artifact due to dental mass (Fig. 1) is not visible in the CT-corrected images with segmentation of transmission data.

**Figure 3:**
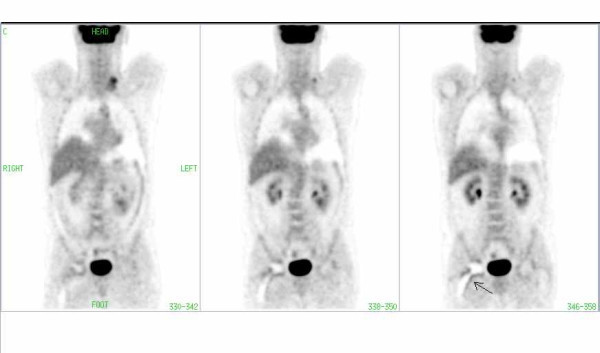
FDG-PET image of a patient with lymphoma involvement in the left cervical region. Note the periprosthetic artifact (arrow) in the region of the right hip in CT corrected images without segmentation.

**Figure 4:**
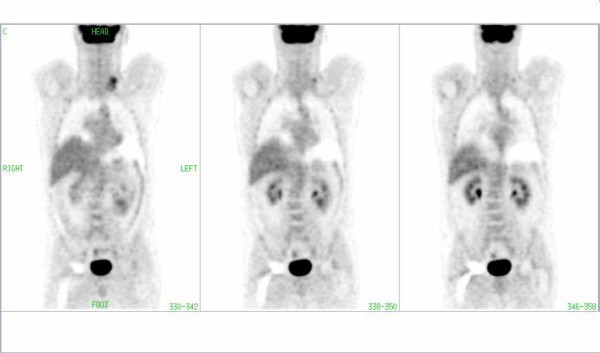
The same patient as in Fig. 3: There is no pathological uptake in the periprosthetic area of the right hip in the CT-corrected images with segmentation of transmission data.

**Figure 5:**
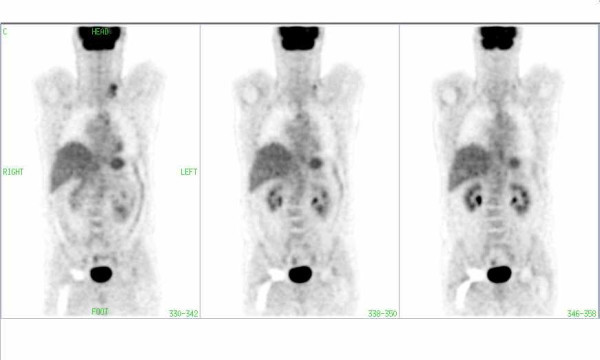
The same patient as in Fig. 3: There is no pathological uptake in the periprosthetic area of the right hip in the images corrected with AC by Cs-137.

The image quality in the area of metal implants was in all cases better with ACseg^+ ^than ACseg^-^, without artifacts seen in images with ACseg^- ^(Figure 1, 2, 3, 4, 5). Further the emission images with ACseg^+ ^were qualitatively comparable to reconstructed data obtained with Cs-137 AC.

As shown in images 1–5 there are hypermetabolic areas seen in the ACseg^- ^images in the area of metal implants which are not visible on ACseg^+ ^reconstructed images. Further we assessed the images without AC. Also in these images the artifacts due to metal implants were not visible, but the image quality was inferior to that of images with both methods of AC.

## Discussion

For better anatomic localization of areas with increased tracer uptake in PET images, anato-metabolic imaging has been proposed during the same session, which has lead to manufacturing combined PET/CT devices [20]. This modality allows to obtain independently high quality PET and CT images as well as to merge both data sets to evaluate the morphological and functional information. For abdominopelvic evaluation, CT has been suggested as procedure of choice with PET imaging [21]. The CT transmission images are at the same time used for purpose of AC which is an indispensable part of image reconstruction and makes it also possible to get quantitative information in the areas of interest. There are three methods to generate attenuation maps from a CT image including segmentation, scaling and dual energy CT scans [22]. Oncologic patients often have artificial metal implants, such as metal braces in the spinal region, chemotherapy ports, dental fillings or implants, pacemaker, that can sometimes be mistaken for other pathologies [23,24]. PET transmission scans using point source of Cs-137 show little or no artifacts. With the significantly higher photon absorption at CT energy, however, these artifacts are present, which are not yet corrected in standard PET-CT protocols. Therefore the reconstruction of emission images without AC has been suggested [22,25]. As we could demonstrate in this study, the artifacts due to metal implants could be avoided using segmentation algorithm for AC with CT data. However, both methods for AC resulted in much better image quality compared to those without AC. In our center we did not make use of contrast medium for the CT part of the examination. But since the artifacts due to contrast medium in PET/CT images [26,27] are caused in the same way as CT hyperintense metal implants, we assume that these would also be avoided using AC with segmentation as described in our study.

## Conclusion

Purpose of this study was to provide a qualitative comparison of attenuation corrected PET images of patients with metal implants produced by a PET-CT device with and without segmentation of CT. It is known that based on reconstruction methods there are differences in quantitative measurements because of the way transmission data are processed [28,29]. Further investigations should be performed to quantitatively estimate the effect of segmentation algorithms used for CT AC.

In case of metal implants, we suggest that PET studies corrected by CT should preferably use the ACseg^+ ^method to avoid the image artifacts.

## Abbreviations

AC = Attenuation correction

Acseg^- ^= AC with non-segmented CT image

Acseg^+ ^= AC with segmented CT image

CT = Computed Tomography

HU = Hounsfield Units

PET = Positron Emission Tomography

## Competing interests

The article-processing charge will be refunded by Philips. Dr Guerchaft is an employee of Philips Medical Systems. All other authors declare that they have no competing interests.

## Authors' contributions

SM performed different reconstruction methods and drafted the manuscript. MG participated in drafting the manuscript and provided technical support. CB carried out the studies and participated in the design of the study. PK provided technical support. MD and PB participated in the design of the study. All authors read and approved the final manuscript.

## Pre-publication history

The pre-publication history for this paper can be accessed here:


